# Intramedullary nailing using K-wires for high-energy distal humeral metaphyseal–diaphyseal fractures accompanying radial nerve palsy in a 2-year-old toddler: A case report

**DOI:** 10.1097/MD.0000000000043322

**Published:** 2025-07-25

**Authors:** Dong-Geun Kang, Dong Hyun Lee, Jin-Hyung Im

**Affiliations:** a Department of Orthopedic Surgery, Gyeongsang National University, College of Medicine and Gyeongsang National University Changwon Hospital, Changwon-si, Republic of Korea; b Department of Orthopedic Surgery, Yeouido St. Mary’s Hospital, College of Medicine, The Catholic University of Korea, Seoul, Republic of Korea.

**Keywords:** distal humeral metaphyseal–diaphyseal fracture, high-energy injury, intramedullary nailing, pediatric open humeral fracture, radial nerve palsy, toddler

## Abstract

**Rationale:**

Pediatric distal humeral diaphyseal fractures are rare and challenging to manage due to anatomical and biomechanical complexities. In addition, humeral shaft fractures in adults are often accompanied by radial nerve paralysis (RNP), but there are no studies on the incidence or treatment of pediatric humeral shaft fractures accompanied by RNP in toddlers. The authors present the outcomes of closed reduction and intramedullary nailing using Kirschner wires (K-wires) for high-energy distal humeral metaphyseal–diaphyseal open fractures accompanied by RNP in a toddler. This case report aims to highlight an effective surgical approach and its outcomes in a toddler, providing insights for clinicians facing similar scenarios.

**Patient concerns:**

A 23-month-old boy was referred to our emergency room after several hospital visits. He complained of pain in his right arm and presented wrist drop due to a crushing injury by a conveyor belt. A 1 cm open wound was located at the lateral side of the elbow.

**Diagnosis:**

The patient was diagnosed with an open displaced fracture in the distal third of the right humerus on radiographic examination and RNP was diagnosed on the basis of physical examination.

**Interventions:**

The authors initially attempted open reduction and internal fixation of the fracture using a Steinmann pin after radial nerve exploration, which confirmed continuity of the normal radial nerve, but fixation failed. Reduction loss and displacement progressed with pin migration the day after surgery, and revision surgery was selected. Closed reduction and intramedullary nailing using K-wires were performed on day 2 after the primary surgery.

**Outcomes:**

The patient recovered his ability to extend the wrist and metacarpophalangeal joint approximately 3 weeks after surgery. At the 1-month and 6-month follow-up, the fracture had healed, and radial nerve function had recovered completely.

**Lessons:**

Intramedullary nailing using K-wires for metaphyseal–diaphyseal fractures of the humerus in toddlers is an effective operative treatment. Among them, high-energy open fractures accompanied by radial nerve palsy might require nerve exploration.

## 1. Introduction

Pediatric humeral shaft fractures are relatively rare, representing 0.4% to 5.4% of all pediatric fractures.^[1,2]^ Metaphyseal–diaphyseal junctional (MDJ) fractures of the distal humerus, which are proximal to the location of humeral condyle fractures, are extremely rare because of their geometric and biomechanical characteristics.^[3]^ Several studies have revealed the treatment methods for and clinical results of treatment for MDJ fractures.^[4–7]^ Adult humeral shaft fractures can be accompanied by radial nerve paralysis (RNP), with an average incidence of 11.8%.^[8]^ In contrast, no studies have revealed the incidence or management of RNP in pediatric patients with humeral shaft fractures, especially toddlers. High-energy injury is rare in the toddler, and the report of MDJ fracture accompanying RNP was few to the best of our knowledge. In this report, we present the outcome of intramedullary (IM) nailing using K-wires for MDJ fractures accompanied by RNP in toddlers and purpose that this report might be helpful in treating similar patients.

## 2. Patient information

A 23-month-old boy suffered a crushing injury to his right elbow as a result of contact with a conveyor belt. A long-arm splint was applied at a private clinic, and he, after visiting other hospitals, was referred to our hospital 1 day after the injury.

### 2.1. Clinical findings

On physical examination, the patient experienced pain and had swelling and a 1 cm open wound on the right elbow, all of which contributed to the loss of both voluntary wrist movement and metacarpophalangeal joint extension. Since he was unable to cooperate with further examination, close observation was necessary.

### 2.2. Diagnostic assessment

**Figure 1.**
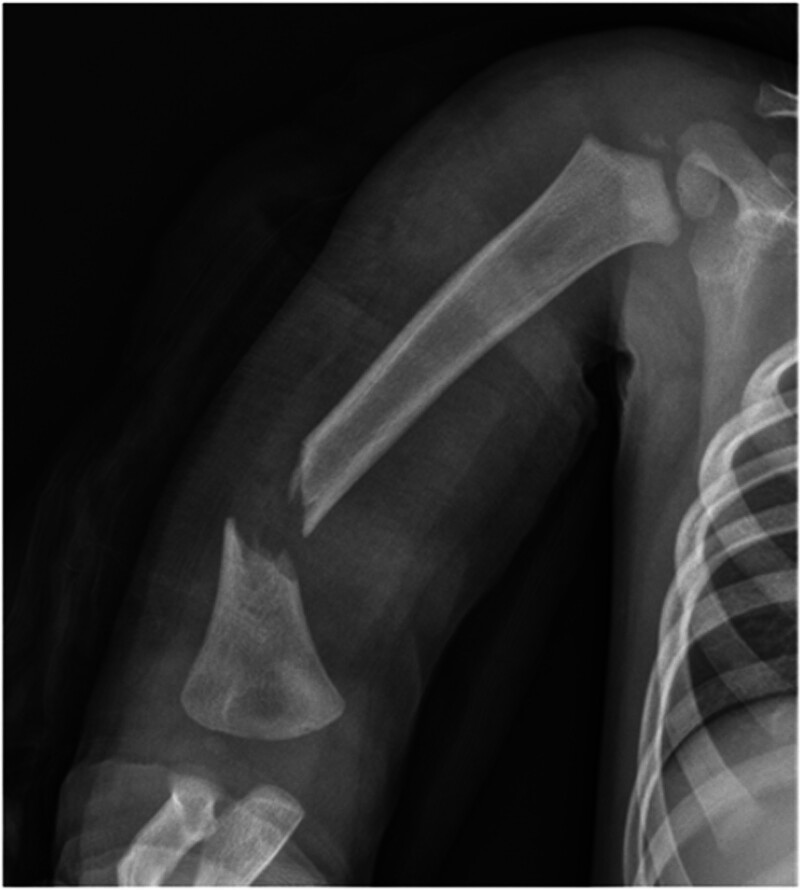
Anteroposterior view of the right humerus showing a severely displaced metaphyseal–diaphyseal fracture of the distal humerus.

**Figure 2.**
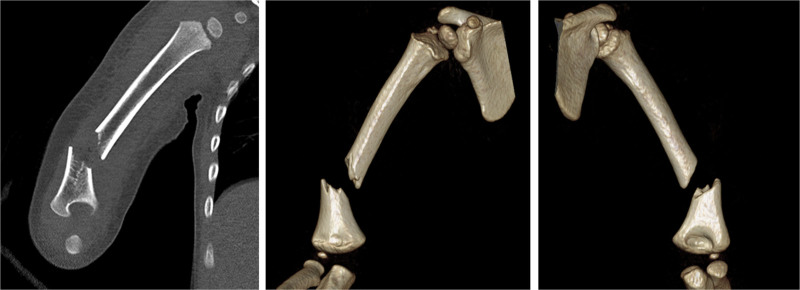
Computed tomography scans of the right humerus revealing a severely displaced metaphyseal–diaphyseal fracture of the distal humerus.

X-ray image of a displaced and angulated fracture at the distal humeral metaphysis–diaphysis. (Fig. 1). The same findings were observed via computed tomography (Fig. 2). Because of the inability to cooperate, ultrasound diagnosis of radial nerve damage was not possible.

### 2.3. Therapeutic intervention

**Figure 3.**
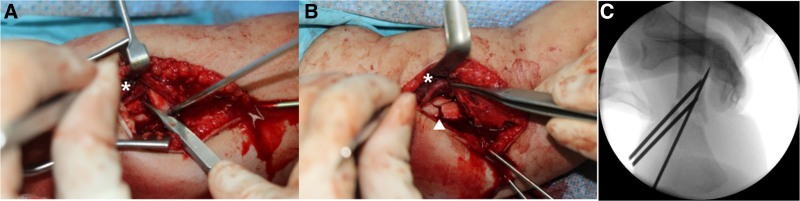
(A) The radial nerve was entrapped between fragments, and it had edema and contusion, but contained normal continuity. (B) The fracture was reduced and fixed with 2.0 mm Steinmann pins. (C) The reduction and fixation status was checked using fluoroscopy (*: radial nerve; white triangle: reduced fragments).

**Figure 4.**
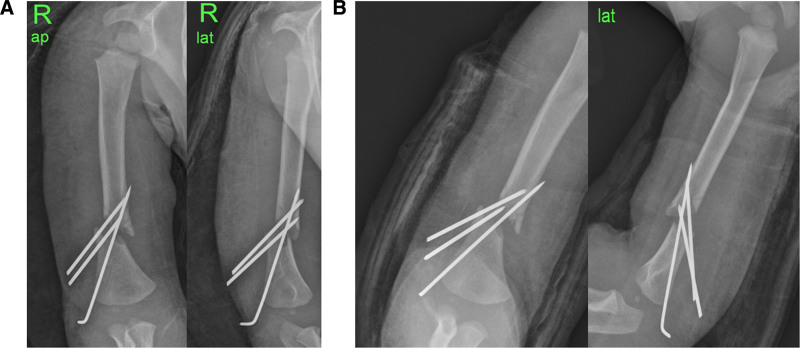
(A) Reduction loss was identified on immediate postoperative radiographs. (B) Increased reduction loss and migration of Steinmann pins were confirmed on radiographs taken the day after the first surgery.

The fracture was not reduced in our emergency room and was remarkably unstable. Surgical treatment was required because of the severe displacement and difficulty maintaining alignment after reduction. With the patient under general anesthesia in the supine position, closed reduction was initially attempted under fluoroscopic guidance. However, acceptable reduction was not possible because the fracture was extremely unstable and irreducible. We made a mini-incision 3 cm proximal to the lateral epicondyle. The fracture site was exposed via meticulous dissection of the brachialis from the triceps. The radial nerve was edematous and contused but maintained normal continuity (Fig. 3A). The fracture was reduced and fixed with 2.0 mm Steinmann pins (Fig. 3B). The reduction and fixation status was checked using fluoroscopy (Fig. 3C.) However, reduction loss was identified on postoperative radiographs (Fig. 3D). The next day, more reduction loss and migration of Steinmann pins were confirmed via X-ray (Fig. 3E). Therefore, a revision operation was needed. With the patient under general anesthesia in the prone position, the Steinmann pins were removed, and closed reduction was attempted under fluoroscopy guidance. After confirming appropriate reduction, 1.4 mm Kirschner wires (K-wires) were inserted through the lateral condyle and the medial condyle for IM nailing (Fig. 4). After reduction, the fixation status and stability were checked under fluoroscopic guidance, and a long-arm splint was applied.

### 2.4. Follow-up and outcomes

**Figure 5.**
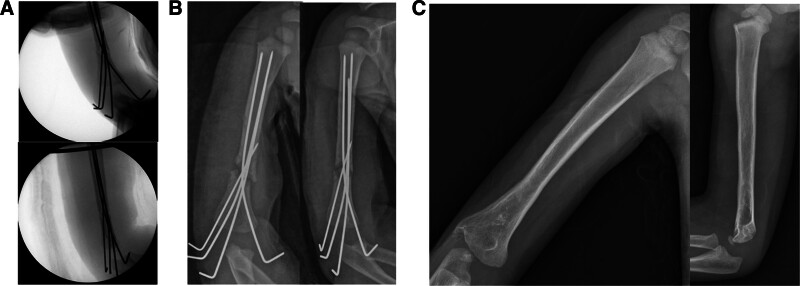
(A) Acceptable reduction was achieved via intramedullary nailing using Kirschner wires according to fluoroscopic images. (B) Postoperative radiographs of the second surgery. (C) Radiographs at 1 year and 6 months after the 2nd surgery.

A long-arm splint was used to immobilize the right upper extremity for 12 weeks postoperatively. At 12 weeks after surgery, the K-wires were removed, and range-of-motion exercises were started. The patient began to recover wrist and metacarpophalangeal joint and wrist joint extension approximately 3 weeks after surgery. Radiographs confirmed complete fracture healing at the 1-month and 6-month follow-ups, with no residual deformity (Fig. 5). A complete radial nerve recovery check was possible with cooperation at the final follow-up.

## 3. Discussion

Although MDJ fractures are very rare, previous studies and case reports have emphasized that they should be treated differently from other distal humerus fractures because of their anatomical characteristics and difficulty in management.^[3–5,7]^ Our case was characterized by younger age, high-energy injury, open fracture, severe displacement, and RNP, among the cases reported in previous studies.

MDJ fractures of the distal humerus are considered more unstable than supracondylar fractures are because the bony shape is closer to a triangle than to the supracondyle and condyle of the humerus. In addition, bone union takes time because of the thin periosteum. However, the distal humerus is stronger than that of the humeral supracondyle because of its thicker cortex and larger bone stock. Thus, MDJ fractures of the distal humerus may be oblique or spiral shaped and break from the thick cortex of the MDJ toward the thin olecranon fossa.^[3,7]^ Owing to their anatomical characteristics, several surgical methods and their successful results have been reported.^[4–7]^ In this case, Steinmann-pin fixation failure occurred after the primary surgery despite open reduction, and retrograde K-wire IM nailing provided stability with closed reduction during revision surgery. A finite element analysis revealed the mechanical stability of various fixation methods according to the MDJ fracture pattern and demonstrated that IM nailing provided optimal rigidity in extension, valgus, and varus loading in a lateral oblique pattern, similar to our case.^[9]^ As with the results of previous studies, the authors consider retrograde IM nailing fixation the first therapeutic choice for unstable displaced MDJ fractures. Afacan et al reported successful outcome of displaced MDJ fracture of the distal humerus treated with IM Steinmann-pin fixation.^[10]^ However, Steinmann pins larger than 2.0 mm are rigid and difficult to support and buttress evenly on both medial and lateral sides of the humerus, so angulation remains after fixation. The authors suggested that flexible K-wires with a diameter of <2.0 mm are effective for balanced fixation.

In most previous studies, surgeries were performed in the supine position. Our first surgery was performed in the supine position in consideration of radial nerve exploration, but the revision surgery was performed in the prone position. As a supracondyle fracture, reduction of rotation is difficult to achieve in the supine position; hence, the prone position might be more suitable for IM nailing MDJ fracture surgery.

Since it is difficult for toddlers to cooperate with physical examination and ultrasound, it is possible to diagnose radial nerve injury through careful observation and a combination of fracture patterns. Our case involved a high-energy open severely displaced Hostein–Lewis fracture, and because it was difficult to communicate with the patient for several months and to carry out conservative treatment, the authors decided on early exploration. Many studies have revealed the treatment of pediatric humeral fractures accompanied by RNP. There have been reports of successful results of conservative treatment of RNP accompanying pediatric humeral shaft fractures.^[11–13]^ On the other hand, nerve grafts have also been used in revision surgeries for distal humerus fractures.^[14]^ While in most studies researchers recommend surgery for RNP after 3 to 6 months of conservative treatment,^[15]^ it is not easy to observe children who are not able to communicate and cooperate for several months. Ito et al reported a case of pediatric supracondylar humerus fracture with radial nerve laceration and concluded that severe placement with complete radial nerve palsy may warrant acute surgical exploration even in the presence of a closed fracture because primary neurorrhaphy may achieve better results than late reconstruction will.^[16]^ Although the authors consider that early exploration of RNP following careful assessment of injury type, fracture pattern, and symptoms in toddlers is feasible, more research is needed. Because this report is a single case study, further studies with larger cohorts are needed to validate these findings.

## 4. Conclusion

MDJ fractures of the distal humerus in children require careful treatment. If surgery is necessary, reduction in the prone position is easier than in the supine position, and sufficient fixation can be achieved by IM fixation with long, flexible K-wires in the medial and lateral directions. Surgical exploration for accompanying RNP requires further research and studies.

## Acknowledgments

This article was edited by a professional English language editing service. Nature Research Editing Service.

## Author contributions

**Data curation:** Dong Hyun Lee.

**Formal analysis:** Dong Hyun Lee.

**Writing – original draft:** Dong-Geun Kang.

**Writing – review & editing:** Jin-Hyung Im.
